# Underlying Causes of Death among Adults with Cerebral Palsy

**DOI:** 10.3390/jcm11216333

**Published:** 2022-10-27

**Authors:** Mark D. Peterson, Allecia M. Wilson, Edward A. Hurvitz

**Affiliations:** 1 Department of Physical Medicine and Rehabilitation, Michigan Medicine, University of Michigan, Ann Arbor, MI 48109, USA; 2 Institute for Healthcare Policy and Innovation, Michigan Medicine, University of Michigan, Ann Arbor, MI 48109, USA; 3 Department of Pathology, Michigan Medicine, University of Michigan, Ann Arbor, MI 48109, USA

**Keywords:** cerebral palsy, epidemiology, mortality, age-adjusted mortality rates, death certificate, medical education

## Abstract

Background: Adults with cerebral palsy (CP) represent a growing population whose healthcare needs are poorly understood. The purpose of this study was to examine trends in the underlying causes of death (UCOD) among adults with CP in the United States. Methods: A national cohort was created from the Centers for Disease Control and Prevention Wide-ranging Online Data for Epidemiologic Research (WONDER) database from 1999 to 2019. The UCOD was determined using the International Statistical Classification of Diseases and Related Health Problems, Tenth Revision (ICD-10 code G80x, Infantile CP) based on death certificate adjudication. Crude and age-adjusted mortality rates (AAMRs), as well as 95% confidence intervals (CIs) were calculated for adults with CP. Results: There were 25,138 deaths where CP was listed as the UCOD between 1999–2019. There was a steady increase in the UCOD attributable to CP in both crude mortality rates and AAMRs, with the highest rates occurring in 2019. The highest co-occurring secondary causes of death were other diseases of the nervous system (e.g., epilepsy), diseases of the respiratory system (e.g., pneumonia), symptoms, signs, and abnormal clinical and laboratory findings, not elsewhere classified (e.g., dysphagia), and diseases of the circulatory system (e.g., cardiovascular disease). Conclusions: Listing the UCOD as CP should be accompanied by other mechanisms leading to mortality in this population.

## 1. Introduction

Cerebral palsy (CP) arises early in life and is viewed as a non-progressive pediatric condition affecting movement, muscle tone, or posture [1]. With improvements in care, individuals with CP live well into late adulthood-prompting a recent paradigm shift to recognize the unique needs of this population across the lifespan [2,3]. Unfortunately, across healthcare systems, adults with CP are often confined to pediatric hospitals and ambulatory care services [4], and they struggle to receive appropriate care, even for the most common disorders such as musculoskeletal comorbidities [5]. As individuals with CP age, they face unique challenges which make their medical care highly complex [6,7,8] and are at risk for dying at younger ages—often from undiagnosed, preventable, noncommunicable diseases [9], and potentially preventable respiratory causes [10].

Mortality records for CP are prone to errors due to the high prevalence of comorbid physical, cognitive, and mental health issues [6,11,12]; however, CP is not a standalone “underlying cause of death” (UCOD) among adults *with* CP. Previous work has demonstrated that postmortem diagnostic overshadowing is common among deceased adults with CP and other neurodevelopmental and intellectual disabilities [13,14,15]. This practice prohibits the identification of the actual medical cause of death and does nothing to inform public health or preventive care efforts to reduce premature mortality. Indeed, the accuracy of the death certificate is vital as it provides important information about the decedent, the circumstances of death, and the true underlying cause(s) of death. Individuals with more severe forms of CP may die in long-term care facilities, in hospice, or at home, resulting in inherent challenges with accurate mortality reporting. The purpose of this study was to examine temporal trends in CP as the UCOD overall and across subgroups stratified by rural-urban area designation in the US. Further, we sought to determine secondary causes of death when CP was listed as the UCOD.

## 2. Methods

We used the US Centers for Disease Control and Prevention Wide-ranging Online Data for Epidemiologic Research (CDC WONDER) database [16]. The Underlying Cause of Death (UCOD) data available on WONDER are county-level national mortality and population data spanning the years 1999–2019. Each death certificate contains a single underlying cause of death, up to 20 additional multiple causes, and demographic data including ethnicity. The number of deaths, crude death rates or age-adjusted death rates, and 95% confidence intervals and standard errors for death rates can be obtained by place of residence (total U.S., region, state, and county), age group (single-year-of age, 5-year age groups, 10-year age groups, and infant age groups), race, Hispanic ethnicity, gender, year, cause-of-death, injury intent, and injury mechanism, drug/alcohol-induced causes, and urbanization categories. Data are also available for the place of death, month and week day of death, and whether an autopsy was performed. For this study, the UCOD was determined using the International Statistical Classification of Diseases and Related Health Problems, Tenth Revision (ICD-10 code G80x, Infantile cerebral palsy) based on death certificate adjudication. Crude mortality rates and 95% confidence intervals were calculated for adults over 20 years of age, and age-adjusted mortality rates (AAMRs) were calculated for adults over 25 years of age between 1999 and 2019. We were limited to doing this because calculations for crude mortality were openly available for all ages, whereas age-adjusted mortality was limited by CDC WONDER to 10-year age bands as follows: (1) <1 year; (2) 1–4 years; (3) 5–14 years; (4) 15–24 years; (5) 25–34 years; (6) 35–44 years; (7) 45–54 years; (8) 55–64 years; (9) 65–74 years; (10) 75–84 years; and (11) 85+ years. The AAMRs were calculated by multiplying the age-specific death rate for each age group by the corresponding weight from the 2000 standard US population, summing across all age groups, and then multiplying by 100,000. Crude mortality and AAMR are expressed per 100,000 population per year.

For the most recent available year (2019), we divided our population using the National Center for Health Statistics urban-rural classification scheme into large metropolitan (≥1 million), medium and small metropolitan (50,000–999,999), and rural (< 50,000) counties per the 2013 US Census classification [17]. Results were further categorized by age (20–34, 35–49, 50–64, and ≥ 65 years), sex, race, and ethnicity. We also examined underlying *and* multiple causes of death with a stepwise approach: when CP was determined as the “underlying cause of death” but there were other causes of mortality in the death certificate, we examined crude mortality of those secondary causes across all possible ICD-10 codes. As data were publicly available and deidentified, ethics committee approval was not required.

## 3. Results

Between 1999 and 2019, there were 25,138 deaths where the UCOD was listed as ICD-10 G80x: Infantile cerebral palsy. There was an increase in deaths attributable to CP in both crude mortality rates and AAMRs from 1999 to 2019, with the highest occurring in 2019 (Table 1).

Most deaths attributed to CP in 2019 occurred in large metropolitan areas (*n* = 866 [45.8%]) followed by medium and small metropolitan areas (*n* = 653 [34.5%]), and rural areas (*n* = 374 [19.7%]); however, the crude mortality rates were significantly higher among lower population areas (Table 2). The majority of deaths attributable to CP occurred among adults that died in a medical facility (*n* = 633 [33.4]), at home (*n* = 588 [31.1%]), or in hospice or a long-term care facility (*n* = 566 [30%]).

There were 6443 unique combinations of multiple causes of death, with CP listed as the UCOD (Table 3). The highest co-occurring causes of death were other diseases of the nervous system (36.4%), diseases of the respiratory system (17.2%), symptoms, signs, and abnormal clinical and laboratory findings, not elsewhere classified (15.3%), and diseases of the circulatory system (8.2%) (individual ICD-10 diseases or causes co-occurring with the primary cause of death [CP] can be found in Supplementary Table S1).

## 4. Discussion

Especially among adults, CP as a standalone UCOD is not sufficient to aid in the understanding of the natural pathophysiology of disease progression. If listed as the UCOD, appropriate mechanisms of death should be listed above it. These other disease mechanisms should clearly represent a causative link directly to CP. If not, CP should not be utilized as the UCOD. Our findings corroborate that of previous studies which have found high rates of mortality attributed to CP, among adults with CP, across developed countries [13,18,19]. According to the current data, the UCOD of more than 25,000 adults with CP were labeled as CP in the US, from 1999 to 2019. While it is certainly understandable that challenges exist with correctly identifying the mechanisms of death in complex medical conditions, labeling a cause of death as CP must be accompanied by other mechanisms leading to death in this population, to bolster our understanding of the natural history of the condition.

Our study also demonstrated numerous secondary/co-occurring causes of death in adults with CP, where CP was listed as the UCOD. The highest co-occurring causes of death were other diseases of the nervous system (e.g., epilepsy), diseases of the respiratory system (e.g., pneumonia), symptoms, signs, and abnormal clinical and laboratory findings, not elsewhere classified (e.g., dysphagia), and diseases of the circulatory system (e.g., cardiovascular disease). By comparison, the most common UCODs for adults without CP in 2019 included (crude mortality rate per 100,000) (#1) diseases of heart (200.8); (#2) malignant neoplasms (182.7); (#3) accidents (52.7); (#4) chronic lower respiratory diseases (47.8); and (#5) cerebrovascular diseases (45.7).

Mortality statistics compiled from death certificates are used to measure health quality, set public health goals and policies, and to direct research and resources. A physician’s principal responsibility in death registration is to complete the medical portion of the death certificate, including the cause of death, according to the Physicians’ Handbook on Medical Certification of Death (available at www.cdc.gov/nchs/data/misc/hb_cod.pdf). The ICD Tenth Revision expanded these coding guidelines in 1999 to be more inclusive, particularly those from indirect causes. We found that a greater proportion of death certificates with CP as the underlying cause of death came from rural areas, and among adults that died at home, in long-term care, or in hospice care. There are unique challenges that face accurate death registration among these patients; however, efforts are needed to facilitate the development of improved clinical screening and rigorous mortality reporting to complete the medical portion of the death certificate for these patients. Several important clinical reporting strategies need to be adopted when an adult with CP dies, including: (1) CP should never be listed as the only cause of death, and (2) CP should not be utilized as the UCOD without the mechanism of death also listed. Limitations include possible errors in coding the cause of death on death certificates and documentation of race/ethnicity. Further, using the CDC WONDER database, we did not have access to individual-level data, which makes it impossible to compute group-based statistical analyses or comparisons to adults without CP. Future research is needed to understand the disparities in mortality rates for CP as the underlying cause of death, between urban and rural areas.

## 5. Conclusions

Given the non-progressive nature of CP, adults with CP may experience a myriad of other diseases that more accurately reflect their true cause of death. CP should not be utilized as the primary UCOD as a default in every patient with CP. This hinders our ability to fully understand the conditions that evolve in adult patients with CP. Between 1999 and 2019, there were more than 25,000 deaths attributed to CP as the underlying cause. Most deaths attributed to CP occurred in large metropolitan areas; however, both crude and age-adjusted mortality rates were significantly higher among rural areas. The highest secondary causes of death were other diseases of the nervous system (e.g., epilepsy), diseases of the respiratory system (e.g., pneumonia), symptoms, signs, and abnormal clinical and laboratory findings (e.g., dysphagia), and diseases of the circulatory system (e.g., cardiovascular disease). Labeling a cause of death as CP must be accompanied by other mechanisms leading to death in this population, to bolster our understanding of the natural history of the condition as well as to increase our understanding of preventable causes of death. Efforts are needed to facilitate the development of improved clinical screening and rigorous death registration to complete the medical portion of the death certificate for this population of patients.

## Figures and Tables

**Table 1:** Trends of “cerebral palsy” (G80x: Infantile cerebral palsy) listed as the underlying cause of death from 1999 to 2019.

Year	Deaths	Population	Crude Rate per 10,000	95% CI	AAMR * per 100,000	95% CI
1999	620	199,000,198	0.3	0.3–0.3	0.3	0.3–0.3
2000	670	200,948,641	0.3	0.3–0.4	0.3	0.3–0.3
2001	727	204,062,414	0.4	0.3–0.4	0.3	0.3–0.3
2002	753	206,451,793	0.4	0.3–0.4	0.3	0.3–0.3
2003	872	208,682,117	0.4	0.4–0.4	0.4	0.4–0.4
2004	936	211,050,944	0.4	0.4–0.5	0.4	0.4–0.5
2005	978	213,511,339	0.5	0.4–0.5	0.4	0.4–0.5
2006	1056	216,055,494	0.5	0.5–0.5	0.4	0.4–0.5
2007	1062	218,481,776	0.5	0.5–0.5	0.5	0.5–0.5
2008	1190	220,975,702	0.5	0.5–0.6	0.5	0.5–0.5
2009	1141	223,491,138	0.5	0.5–0.5	0.5	0.5–0.5
2010	1221	225,477,982	0.5	0.5–0.6	0.5	0.5–0.5
2011	1230	228,746,768	0.5	0.5–0.6	0.5	0.5–0.6
2012	1259	231,409,240	0.5	0.5–0.6	0.5	0.5–0.5
2013	1418	233,880,752	0.6	0.6–0.6	0.6	0.5–0.6
2014	1429	236,721,454	0.6	0.6–0.6	0.6	0.5–0.6
2015	1555	239,293,130	0.6	0.6–0.7	0.6	0.6–0.7
2016	1622	241,022,445	0.7	0.6–0.7	0.7	0.6–0.7
2017	1696	243,565,966	0.7	0.7–0.7	0.7	0.6–0.7
2018	1810	245,184,769	0.7	0.7–0.8	0.7	0.7–0.8
2019	1893	246,614,107	0.8	0.7–0.8	0.8	0.7–0.8
Total	25,138					

* AMMRs—age-adjusted mortality rates; CI—confidence intervals; AMMRs were calculated for adults over 25 years.

**Table 2:** Adults with cerebral palsy who died in 2019, with the underlying cause of death listed as “cerebral palsy”.

	Deaths	Population	Crude Rate per 100,000	95% CI
All Ages	2168	328,239,523	0.7	0.6–0.7
Adults	1893	246,614,107	0.8	0.7–0.8
Age Categories				
20–34 years	479	67,573,261	0.7	0.6–0.8
35–49 years	331	44,168,826	0.7	0.7–0.8
40–49 years	408	62,056,895	0.7	0.6–0.7
50–64 years	498	62,925,688	0.8	0.7–0.9
≥65 years	508	54,058,263	0.9	0.9–1.0
Gender				
Female	849	126,652,620	0.7	0.6–0.7
Male	1044	119,961,487	0.9	0.8–0.9
Race/Ethnicity				
White	1555	193,833,509	0.8	0.8–0.8
Black	301	32,937,597	0.9	0.8–1.0
Asian	26	16,590,321	0.2	0.1–0.2
Hispanic	187	39,870,392	0.5	0.4–0.5
Other	11	3,252,680	n/a	n/a
Urbanization				
Metro (Large)	866	137,873,755	0.6	0.6–0.7
Metro (Medium and Small)	653	73,879,878	0.9	0.8–1.0
Non-Metro (Rural)	374	34,860,474	1.1	1.01–1.2

**Table 3:** Adults with cerebral palsy who died in 2019, with the underlying cause of death listed as “cerebral palsy”, and other multiple causes of death.

Population *n* = 246,614,107	Deaths	Crude Rate per 100,000	95% CI
A00-B99 (Certain infectious and parasitic diseases)	205	0.1	0.1–0.1
C00-D48 (Neoplasms)	20	0.0	0.0–0.0
D50-D89 (Diseases of the blood and blood-forming organs and certain disorders involving the immune mechanism)	34	0.0	0.0–0.0
E00-E89 (Endocrine, nutritional, and metabolic diseases)	229	0.1	0.1–0.1
F01-F99 (Mental and behavioral disorders)	154	0.1	0.1–0.1
G00-G98 (Diseases of the nervous system)	2344	1.0	0.9–1.0
H00-H59 (Diseases of the eye and adnexa)	5	n/a	n/a
I00-I99 (Diseases of the circulatory system)	528	0.2	0.2–0.2
J00-J98 (Diseases of the respiratory system)	1110	0.5	0.4–0.5
K00-K92 (Diseases of the digestive system)	154	0.1	0.1–0.1
L00-L98 (Diseases of the skin and subcutaneous tissue)	41	0.0	0.0–0.0
M00-M99 (Diseases of the musculoskeletal system and connective tissue)	58	0.0	0.0–0.0
N00-N99 (Diseases of the genitourinary system)	165	0.1	0.1–0.1
P00-P96 (Certain conditions originating in the perinatal period)	7	n/a	n/a
Q00-Q99 (Congenital malformations, deformations, and chromosomal abnormalities)	29	0.0	0.0–0.0
R00-R99 (Symptoms, signs, and abnormal clinical and laboratory findings, not elsewhere classified)	984	0.4	0.4–0.4
S00-T98 (Injury, poisoning, and certain other consequences of external causes)	170	0.1	0.1–0.1
V01-Y89 (External causes of morbidity and mortality)	202	0.1	0.1–0.1
Y88.3 (Sequelae of surgical and medical procedures as the cause of abnormal reaction of the patient, or of later complication, without mention of misadventure at the time of the procedure)	4	n/a	n/a

## Data Availability

Data are publicly available through the US Centers for Disease Control and Prevention Wide-ranging Online Data for Epidemiologic Research (CDC WONDER) database: https://wonder.cdc.gov/Deaths-by-Underlying-Cause.html.

## Supplementary Materials

The following are available online at https://www.mdpi.com/article/10.3390/jcm11216333/s1, Table S1: Adults that died in 2019, with the underlying cause of death listed as “cerebral palsy”, and 6443 unique combinations of multiple causes of death.
