# Supplementary material for: Underlying Causes of Death among Adults with Cerebral Palsy
Source: J Clin Med. 2022 Oct 27;11(21):6333. doi: 10.3390/jcm11216333 (PMC9656674; doi:10.3390/jcm11216333)
Supplement: Supplementary file 1 [file jcm-11-06333-s001.zip › jcm-1964870-supplementary.pdf]

Supplemental Table S1. Adults that died in 2019, with the underlying cause of death listed as "cerebral palsy", and 6443 unique combinations of multiple causes of death.

Population n=246,614,107	Deaths	Crude Rate Per 100,000	95% CI
A00-B99 (Certain infectious and parasitic diseases)	205	0.1	0.1-0.1
A04.7 (Enterocolitis due to Clostridium difficile)	5		
A09.9 (Gastroenteritis and colitis of unspecified origin)	2		
A16.9 (Respiratory tuberculosis unspecified, without mention of bacteriological or histological confirmation)	1		
A41.4 (Septicaemia due to anaerobes)	1		
A41.5 (Septicaemia due to other Gram-negative organisms)	1		
A41.9 (Septicaemia, unspecified)	174		
A49.0 (Staphylococcal infection, unspecified)	2		
A49.1 (Streptococcal infection, unspecified)	1		
A49.8 (Other bacterial infections of unspecified site)	3		
A49.9 (Bacterial infection, unspecified)	6		
B25.9 (Cytomegaloviral disease, unspecified)	1		
B49 (Unspecified mycosis)	1		
B59 (Pneumocystosis)	1		
B91 (Sequelae of poliomyelitis)	2		
B94.8 (Sequelae of other specified infectious and parasitic diseases)	3		
B99 (Other and unspecified infectious diseases)	1		
C00-D48 (Neoplasms)	20	0.0	0-0
C02.9 (Tongue, unspecified - Malignant neoplasms)	1		
C18.2 (Ascending colon - Malignant neoplasms)	1		
C18.9 (Colon, unspecified - Malignant neoplasms)	2		
C22.0 (Liver cell carcinoma - Malignant neoplasms)	1		
C44.3 (Skin of other and unspecified parts of face - Malignant neoplasms)	1		
C50.9 (Breast, unspecified - Malignant neoplasms)	3		
C61 (Malignant neoplasm of prostate)	2		
C64 (Malignant neoplasm of kidney, except renal pelvis)	1		
C76.3 (Pelvis - Malignant neoplasms)	1		
C80 (Malignant neoplasm without specification of site)	1		
C95.9 (Leukaemia, unspecified - Malignant neoplasms)	1		
C96.9 (Malignant neoplasm of lymphoid, haematopoietic and related tissue, unspecified - Malignant neoplasms)	1		
D32.0 (Cerebral meninges - Benign neoplasms)	1		

D40.1 (Testis - Uncertain neoplasms)	1		
D43.2 (Brain, unspecified - Uncertain neoplasms)	1		
D46.9 (Myelodysplastic syndrome, unspecified - Uncertain neoplasms)	1		
D50-D89 (Diseases of the blood and blood-forming organs and certain disorders involving the immune mechanism)	34	0.0	0-0
D50.0 (Iron deficiency anaemia secondary to blood loss (chronic))	1		
D50.9 (Iron deficiency anaemia, unspecified)	1		
D56.9 (Thalassaemia, unspecified)	1		
D59.9 (Acquired haemolytic anaemia, unspecified)	1		
D61.9 (Aplastic anaemia, unspecified)	2		
D62 (Acute posthaemorrhagic anaemia)	1		
D64.9 (Anaemia, unspecified)	20		
D65 (Disseminated intravascular coagulation [defibrination syndrome])	1		
D69.3 (Idiopathic thrombocytopenic purpura)	1		
D69.4 (Other primary thrombocytopenia)	1		
D70 (Agranulocytosis)	1		
D73.8 (Other diseases of spleen)	1		
D75.8 (Other specified diseases of blood and blood-forming organs)	1		
D86.9 (Sarcoidosis, unspecified)	1		
E00-E89 (Endocrine, nutritional and metabolic diseases)	229	0.1	0.1-0.1
E03.9 (Hypothyroidism, unspecified)	19		
E06.3 (Autoimmune thyroiditis)	1		
E10.9 (Insulin-dependent diabetes mellitus, without complications)	1		
E11.2 (Non-insulin-dependent diabetes mellitus, with renal complications)	1		
E11.4 (Non-insulin-dependent diabetes mellitus, with neurological complications)	1		
E11.7 (Non-insulin-dependent diabetes mellitus, with multiple complications)	1		
E11.9 (Non-insulin-dependent diabetes mellitus, without complications)	16		
E14.2 (Unspecified diabetes mellitus, with renal complications)	2		
E14.5 (Unspecified diabetes mellitus, with peripheral circulatory complications)	1		
E14.9 (Unspecified diabetes mellitus, without complications)	25		
E16.2 (Hypoglycaemia, unspecified)	2		
E23.0 (Hypopituitarism)	1		
E27.4 (Other and unspecified adrenocortical insufficiency)	3		
E43 (Unspecified severe protein-energy malnutrition)	12		
E44.0 (Moderate protein-energy malnutrition)	1		

E45 (Retarded development following protein-energy malnutrition)	2		
E46 (Unspecified protein-energy malnutrition)	57		
E63.9 (Nutritional deficiency, unspecified)	2		
E66.8 (Other obesity)	3		
E66.9 (Obesity, unspecified)	8		
E70.1 (Other hyperphenylalaninaemias)	1		
E71.3 (Disorders of fatty-acid metabolism)	1		
E72.2 (Disorders of urea cycle metabolism)	1		
E75.2 (Other sphingolipidosis)	1		
E76.2 (Other mucopolysaccharidoses)	1		
E77.1 (Defects in glycoprotein degradation)	1		
E78.0 (Pure hypercholesterolaemia)	1		
E78.5 (Hyperlipidaemia, unspecified)	16		
E78.9 (Disorder of lipoprotein metabolism, unspecified)	2		
E79.1 (Lesch-Nyhan syndrome)	1		
E84.8 (Cystic fibrosis with other manifestations)	1		
E86 (Volume depletion)	15		
E87.0 (Hyperosmolality and hypernatraemia)	5		
E87.1 (Hypo-osmolality and hyponatraemia)	5		
E87.2 (Acidosis)	6		
E87.5 (Hyperkalaemia)	2		
E87.6 (Hypokalaemia)	2		
E87.7 (Fluid overload)	1		
E87.8 (Other disorders of electrolyte and fluid balance, not elsewhere classified)	2		
E88.9 (Metabolic disorder, unspecified)	5		
F01-F99 (Mental and behavioural disorders)	154	0.1	0.1-0.1
F01.9 (Vascular dementia, unspecified)	5		
F03 (Unspecified dementia)	16		
F09 (Unspecified organic or symptomatic mental disorder)	1		
F17.9 (Mental and behavioural disorders due to use of tobacco, unspecified mental and behavioural disorder)	11		
F19.9 (Mental and behavioural disorders due to multiple drug use and use of other psychoactive substances, unspecified mental and behavioural disorder)	1		
F20.0 (Paranoid schizophrenia)	1		
F20.9 (Schizophrenia, unspecified)	7		
F25.9 (Schizoaffective disorder, unspecified)	3		
F29 (Unspecified nonorganic psychosis)	1		
F31.9 (Bipolar affective disorder, unspecified)	7		
F32.2 (Severe depressive episode without psychotic symptoms)	3		
F32.9 (Depressive episode, unspecified)	8		

F40.0 (Agoraphobia)	1		
F41.9 (Anxiety disorder, unspecified)	6		
F45.9 (Somatoform disorder, unspecified)	1		
F71 (Moderate mental retardation)	2		
F72 (Severe mental retardation)	6		
F73 (Profound mental retardation)	5		
F79 (Unspecified mental retardation)	52		
F81.9 (Developmental disorder of scholastic skills, unspecified)	6		
F84.0 (Childhood autism)	5		
F89 (Unspecified disorder of psychological development)	3		
F91.2 (Socialized conduct disorder)	1		
F91.9 (Conduct disorder, unspecified)	1		
F95.2 (Combined vocal and multiple motor tic disorder [de la Tourette])	1		
G00-G98 (Diseases of the nervous system)	2344	1.0	0.9-1.0
G03.9 (Meningitis, unspecified)	1		
G04.9 (Encephalitis, myelitis and encephalomyelitis, unspecified)	1		
G09 (Sequelae of inflammatory diseases of central nervous system)	1		
G11.1 (Early-onset cerebellar ataxia)	1		
G14 (Postpolio syndrome)	1		
G20 (Parkinson disease)	5		
G24.9 (Dystonia, unspecified)	2		
G25.0 (Essential tremor)	1		
G30.9 (Alzheimer disease, unspecified)	5		
G31.9 (Degenerative disease of nervous system, unspecified)	2		
G35 (Multiple sclerosis)	2		
G40.3 (Generalized idiopathic epilepsy and epileptic syndromes)	3		
G40.4 (Other generalized epilepsy and epileptic syndromes)	7		
G40.6 (Grand mal seizures, unspecified (with or without petit mal))	2		
G40.8 (Other epilepsy)	1		
G40.9 (Epilepsy, unspecified)	110		
G41.9 (Status epilepticus, unspecified)	22		
G43.9 (Migraine, unspecified)	2		
G47.3 (Sleep apnoea)	13		
G70.9 (Myoneural disorder, unspecified)	2		
G71.0 (Muscular dystrophy)	2		
G71.1 (Myotonic disorders)	2		
G80.0 (Spastic cerebral palsy)	42		

G80.1 (Spastic diplegia)	24		
G80.2 (Infantile hemiplegia)	16		
G80.3 (Dyskinetic cerebral palsy)	4		
G80.4 (Ataxic cerebral palsy)	3		
G80.8 (Other infantile cerebral palsy)	87		
G80.9 (Infantile cerebral palsy, unspecified)	1802		
G81.0 (Flaccid hemiplegia)	1		
G81.1 (Spastic hemiplegia)	2		
G81.9 (Hemiplegia, unspecified)	7		
G82.1 (Spastic paraplegia)	1		
G82.2 (Paraplegia, unspecified)	7		
G82.4 (Spastic tetraplegia)	25		
G82.5 (Tetraplegia, unspecified)	42		
G83.0 (Diplegia of upper limbs)	1		
G83.1 (Monoplegia of lower limb)	2		
G83.9 (Paralytic syndrome, unspecified)	1		
G90.8 (Other disorders of autonomic nervous system)	1		
G91.1 (Obstructive hydrocephalus)	2		
G91.9 (Hydrocephalus, unspecified)	13		
G93.1 (Anoxic brain damage, not elsewhere classified)	48		
G93.4 (Encephalopathy, unspecified)	20		
G93.6 (Cerebral oedema)	2		
G93.7 (Reye syndrome)	1		
G93.8 (Other specified disorders of brain)	1		
G97.8 (Other postprocedural disorders of nervous system)	1		
H00-H59 (Diseases of the eye and adnexa)	5		
H40.9 (Glaucoma, unspecified)	1		
H54.0 (Blindness, both eyes)	4		
I00-I99 (Diseases of the circulatory system)	528	0.2	0.2-0.2
I10 (Essential (primary) hypertension)	83		
I11.9 (Hypertensive heart disease without (congestive) heart failure)	10		
I12.0 (Hypertensive renal disease with renal failure)	5		
I21.4 (Acute subendocardial myocardial infarction)	2		
I21.9 (Acute myocardial infarction, unspecified)	5		
I25.0 (Atherosclerotic cardiovascular disease, so described)	3		
I25.1 (Atherosclerotic heart disease)	10		
I25.9 (Chronic ischaemic heart disease, unspecified)	1		
I26.9 (Pulmonary embolism without mention of acute cor pulmonale)	8		

I27.2 (Other secondary pulmonary hypertension)	1		
I31.3 (Pericardial effusion (noninflammatory))	2		
I33.0 (Acute and subacute infective endocarditis)	1		
I42.9 (Cardiomyopathy, unspecified)	1		
I44.2 (Atrioventricular block, complete)	1		
I45.8 (Other specified conduction disorders)	1		
I46.1 (Sudden cardiac death, so described)	4		
I46.9 (Cardiac arrest, unspecified)	247		
I47.1 (Supraventricular tachycardia)	2		
I48 (Atrial fibrillation and flutter)	16		
I49.0 (Ventricular fibrillation and flutter)	1		
I49.9 (Cardiac arrhythmia, unspecified)	20		
I50.0 (Congestive heart failure)	32		
I50.1 (Left ventricular failure)	1		
I50.9 (Heart failure, unspecified)	13		
I51.6 (Cardiovascular disease, unspecified)	2		
I51.7 (Cardiomegaly)	1		
I51.8 (Other ill-defined heart diseases)	2		
I51.9 (Heart disease, unspecified)	3		
I61.3 (Intracerebral haemorrhage in brain stem)	2		
I62.0 (Subdural haemorrhage (acute)(nontraumatic))	3		
I63.9 (Cerebral infarction, unspecified)	1		
I64 (Stroke, not specified as haemorrhage or infarction)	10		
I67.8 (Other specified cerebrovascular diseases)	1		
I67.9 (Cerebrovascular disease, unspecified)	3		
I69.1 (Sequelae of intracerebral haemorrhage)	1		
I69.2 (Sequelae of other nontraumatic intracranial haemorrhage)	1		
I69.3 (Sequelae of cerebral infarction)	2		
I69.4 (Sequelae of stroke, not specified as haemorrhage or infarction)	3		
I69.8 (Sequelae of other and unspecified cerebrovascular diseases)	1		
I73.9 (Peripheral vascular disease, unspecified)	6		
I80.2 (Phlebitis and thrombophlebitis of other deep vessels of lower extremities)	4		
I82.8 (Embolism and thrombosis of other specified veins)	1		
I82.9 (Embolism and thrombosis of unspecified vein)	1		
I83.0 (Varicose veins of lower extremities with ulcer)	1		
I95.9 (Hypotension, unspecified)	9		
J00-J98 (Diseases of the respiratory system)	1110	0.5	0.4-0.5
J00 (Acute nasopharyngitis [common cold])	1		
J04.1 (Acute tracheitis)	1		

J10.0 (Influenza with pneumonia, influenza virus identified)	3
J10.1 (Influenza with other respiratory manifestations, influenza virus identified)	1
J12.9 (Viral pneumonia, unspecified)	2
J13 (Pneumonia due to Streptococcus pneumoniae)	2
J15.0 (Pneumonia due to Klebsiella pneumoniae)	1
J15.1 (Pneumonia due to Pseudomonas)	3
J15.2 (Pneumonia due to staphylococcus)	5
J15.9 (Bacterial pneumonia, unspecified)	11
J18.0 (Bronchopneumonia, unspecified)	5
J18.1 (Lobar pneumonia, unspecified)	3
J18.2 (Hypostatic pneumonia, unspecified)	2
J18.9 (Pneumonia, unspecified)	202
J21.9 (Acute bronchiolitis, unspecified)	1
J36 (Peritonsillar abscess)	1
J39.8 (Other specified diseases of upper respiratory tract)	3
J40 (Bronchitis, not specified as acute or chronic)	4
J42 (Unspecified chronic bronchitis)	1
J43.9 (Emphysema, unspecified)	2
J44.0 (Chronic obstructive pulmonary disease with acute lower respiratory infection)	4
J44.1 (Chronic obstructive pulmonary disease with acute exacerbation, unspecified)	1
J44.8 (Other specified chronic obstructive pulmonary disease)	1
J44.9 (Chronic obstructive pulmonary disease, unspecified)	27
J45.9 (Asthma, unspecified)	17
J47 (Bronchiectasis)	2
J69.0 (Pneumonitis due to food and vomit)	298
J80 (Adult respiratory distress syndrome)	8
J84.1 (Other interstitial pulmonary diseases with fibrosis)	2
J84.9 (Interstitial pulmonary disease, unspecified)	1
J86.9 (Pyothorax without fistula)	4
J90 (Pleural effusion, not elsewhere classified)	3
J93.9 (Pneumothorax, unspecified)	1
J94.8 (Other specified pleural conditions)	1
J95.0 (Tracheostomy malfunction)	1
J95.8 (Other postprocedural respiratory disorders)	3
J96.0 (Acute respiratory failure)	163
J96.1 (Chronic respiratory failure)	89
J96.9 (Respiratory failure, unspecified)	204
J98.1 (Pulmonary collapse)	3
J98.4 (Other disorders of lung)	15

J98.8 (Other specified respiratory disorders)	5		
J98.9 (Respiratory disorder, unspecified)	3		
K00-K92 (Diseases of the digestive system)	154	0.1	0.1-0.1
K05.1 (Chronic gingivitis)	1		
K21.0 (Gastro-oesophageal reflux disease with oesophagitis)	1		
K21.9 (Gastro-oesophageal reflux disease without oesophagitis)	21		
K22.1 (Ulcer of oesophagus)	1		
K22.2 (Oesophageal obstruction)	3		
K22.4 (Dyskinesia of oesophagus)	2		
K22.6 (Gastro-oesophageal laceration-haemorrhage syndrome)	1		
K22.7 (Barrett esophagus)	3		
K22.8 (Other specified diseases of oesophagus)	1		
K25.4 (Gastric ulcer, chronic or unspecified with haemorrhage)	1		
K26.4 (Duodenal ulcer, chronic or unspecified with haemorrhage)	1		
K26.6 (Duodenal ulcer, chronic or unspecified with both haemorrhage and perforation)	1		
K27.4 (Peptic ulcer, site unspecified, chronic or unspecified with haemorrhage)	1		
K27.9 (Peptic ulcer, site unspecified, unspecified as acute or chronic, without haemorrhage or perforation)	1		
K29.5 (Chronic gastritis, unspecified)	1		
K29.7 (Gastritis, unspecified)	1		
K31.6 (Fistula of stomach and duodenum)	2		
K31.8 (Other specified diseases of stomach and duodenum)	4		
K44.9 (Diaphragmatic hernia without obstruction or gangrene)	5		
K50.9 (Crohn disease, unspecified)	4		
K51.9 (Ulcerative colitis, unspecified)	1		
K52.9 (Noninfective gastroenteritis and colitis, unspecified)	1		
K55.0 (Acute vascular disorders of intestine)	1		
K55.9 (Vascular disorder of intestine, unspecified)	3		
K56.0 (Paralytic ileus)	2		
K56.2 (Volvulus)	2		
K56.3 (Gallstone ileus)	1		
K56.4 (Other impaction of intestine)	1		
K56.6 (Other and unspecified intestinal obstruction)	16		
K56.7 (Ileus, unspecified)	8		
K58.9 (Irritable bowel syndrome without diarrhoea)	1		
K59.0 (Constipation)	11		
K59.3 (Megacolon, not elsewhere classified)	3		

K59.9 (Functional intestinal disorder, unspecified)	1		
K62.9 (Disease of anus and rectum, unspecified)	1		
K63.1 (Perforation of intestine (nontraumatic))	5		
K63.2 (Fistula of intestine)	2		
K63.8 (Other specified diseases of intestine)	1		
K63.9 (Disease of intestine, unspecified)	3		
K65.0 (Acute peritonitis)	2		
K65.9 (Peritonitis, unspecified)	2		
K74.6 (Other and unspecified cirrhosis of liver)	1		
K76.0 (Fatty (change of) liver, not elsewhere classified)	1		
K76.9 (Liver disease, unspecified)	2		
K80.2 (Calculus of gallbladder without cholecystitis)	1		
K80.5 (Calculus of bile duct without cholangitis or cholecystitis)	1		
K81.9 (Cholecystitis, unspecified)	3		
K82.9 (Disease of gallbladder, unspecified)	1		
K85.9 (Acute pancreatitis, unspecified)	3		
K90.4 (Malabsorption due to intolerance, not elsewhere classified)	1		
K91.3 (Postoperative intestinal obstruction)	1		
K91.8 (Other postprocedural disorders of digestive system, not elsewhere classified)	2		
K92.1 (Melaena)	2		
K92.2 (Gastrointestinal haemorrhage, unspecified)	10		
K92.8 (Other specified diseases of digestive system)	1		
L00-L98 (Diseases of the skin and subcutaneous tissue)	41	0.0	0.0-0.0
L03.9 (Cellulitis, unspecified)	3		
L08.9 (Local infection of skin and subcutaneous tissue, unspecified)	1		
L40.5 (Arthropathic psoriasis)	1		
L89.1 (Stage II decubitus ulcer)	1		
L89.2 (Stage III decubitus ulcer)	2		
L89.3 (Stage IV decubitus ulcer)	7		
L89.9 (Decubitus ulcer and pressure area, unspecified)	20		
L97 (Ulcer of lower limb, not elsewhere classified)	1		
L98.4 (Chronic ulcer of skin, not elsewhere classified)	3		
L98.8 (Other specified disorders of skin and subcutaneous tissue)	2		
M00-M99 (Diseases of the musculoskeletal system and connective tissue)	58	0.0	0.0-0.0
M19.9 (Arthrosis, unspecified)	3		
M24.5 (Contracture of joint)	2		
M25.9 (Joint disorder, unspecified)	1		
M41.0 (Infantile idiopathic scoliosis)	1		

M41.4 (Neuromuscular scoliosis)	6		
M41.8 (Other forms of scoliosis)	1		
M41.9 (Scoliosis, unspecified)	22		
M43.2 (Other fusion of spine)	1		
M46.2 (Osteomyelitis of vertebra)	1		
M47.8 (Other spondylosis)	1		
M48.0 (Spinal stenosis)	2		
M62.3 (Immobility syndrome (paraplegic))	1		
M62.5 (Muscle wasting and atrophy, not elsewhere classified)	4		
M62.8 (Other specified disorders of muscle)	3		
M79.7 (Fibromyalgia)	1		
M81.9 (Osteoporosis, unspecified)	3		
M85.8 (Other specified disorders of bone density and structure)	1		
M86.9 (Osteomyelitis, unspecified)	4		
N00-N99 (Diseases of the genitourinary system)	165	0.1	0.1-0.1
N12 (Tubulo-interstitial nephritis, not specified as acute or chronic)	1		
N13.0 (Hydronephrosis with ureteropelvic junction obstruction)	1		
N13.3 (Other and unspecified hydronephrosis)	2		
N13.9 (Obstructive and reflux uropathy, unspecified)	2		
N17.9 (Acute renal failure, unspecified)	16		
N18.3 (Chronic kidney disease, stage 3)	1		
N18.5 (Chronic kidney disease, stage 5)	2		
N18.9 (Chronic renal failure, unspecified)	10		
N19 (Unspecified renal failure)	10		
N20.0 (Calculus of kidney)	7		
N20.9 (Urinary calculus, unspecified)	1		
N28.8 (Other specified disorders of kidney and ureter)	13		
N28.9 (Disorder of kidney and ureter, unspecified)	2		
N31.9 (Neuromuscular dysfunction of bladder, unspecified)	18		
N32.8 (Other specified disorders of bladder)	1		
N39.0 (Urinary tract infection, site not specified)	74		
N39.9 (Disorder of urinary system, unspecified)	1		
N40 (Hyperplasia of prostate)	3		
P00-P96 (Certain conditions originating in the perinatal period)	7		n/a
P07.2 (Extreme immaturity)	2		
P07.3 (Other preterm infants)	2		
P15.9 (Birth injury, unspecified)	1		
P90 (Convulsions of newborn)	2		

Q00-Q99 (Congenital malformations, deformations and chromosomal abnormalities)	29	0.0	0.0-0.0
Q24.0 (Dextrocardia)	1		
Q24.9 (Congenital malformation of heart, unspecified)	1		
Q25.6 (Stenosis of pulmonary artery)	1		
Q32.1 (Other congenital malformations of trachea)	1		
Q39.0 (Atresia of oesophagus without fistula)	1		
Q60.0 (Renal agenesis, unilateral)	1		
Q64.7 (Other congenital malformations of bladder and urethra)	1		
Q65.8 (Other congenital deformities of hip)	1		
Q79.4 (Prune belly syndrome)	1		
Q79.6 (Ehlers-Danlos syndrome)	1		
Q82.3 (Incontinentia pigmenti)	1		
Q82.4 (Ectodermal dysplasia (anhidrotic))	1		
Q85.0 (Neurofibromatosis (nonmalignant))	1		
Q85.1 (Tuberous sclerosis)	1		
Q86.0 (Fetal alcohol syndrome (dysmorphic))	2		
Q87.0 (Congenital malformation syndromes predominantly affecting facial appearance)	1		
Q87.1 (Congenital malformation syndromes predominantly associated with short stature)	1		
Q89.7 (Multiple congenital malformations, not elsewhere classified)	1		
Q89.9 (Congenital malformation, unspecified)	1		
Q90.9 (Down syndrome, unspecified)	5		
Q93.5 (Other deletions of part of a chromosome)	2		
Q93.9 (Deletion from autosomes, unspecified)	1		
Q99.9 (Chromosomal abnormality, unspecified)	1		
R00-R99 (Symptoms, signs and abnormal clinical and laboratory findings, not elsewhere classified)	984	0.4	0.4-0.4
R00.0 (Tachycardia, unspecified)	2		
R00.1 (Bradycardia, unspecified)	2		
R01.1 (Cardiac murmur, unspecified)	2		
R02 (Gangrene, not elsewhere classified)	1		
R06.0 (Dyspnoea)	4		
R06.4 (Hyperventilation)	3		
R06.8 (Other and unspecified abnormalities of breathing)	18		
R09.0 (Asphyxia)	52		
R09.2 (Respiratory arrest)	64		
R10.4 (Other and unspecified abdominal pain)	2		
R11 (Nausea and vomiting)	10		
R13 (Dysphagia)	205		
R15 (Faecal incontinence)	1		

R18 (Ascites)	2		
R19.8 (Other specified symptoms and signs involving the digestive system and abdomen)	1		
R25.2 (Cramp and spasm)	5		
R26.3 (Immobility)	16		
R27.0 (Ataxia, unspecified)	2		
R27.8 (Other and unspecified lack of coordination)	1		
R29.3 (Abnormal posture)	1		
R29.8 (Other and unspecified symptoms and signs involving the nervous and musculoskeletal systems)	11		
R32 (Unspecified urinary incontinence)	1		
R33 (Retention of urine)	4		
R41.8 (Other and unspecified symptoms and signs involving cognitive functions and awareness)	7		
R45.1 (Restlessness and agitation)	1		
R47.0 (Dysphasia and aphasia)	10		
R47.8 (Other and unspecified speech disturbances)	1		
R50.9 (Fever, unspecified)	2		
R51 (Headache)	1		
R52.2 (Other chronic pain)	1		
R53 (Malaise and fatigue)	29		
R54 (Senility)	7		
R56.8 (Other and unspecified convulsions)	309		
R57.0 (Cardiogenic shock)	3		
R57.8 (Other shock)	1		
R57.9 (Shock, unspecified)	6		
R58 (Haemorrhage, not elsewhere classified)	2		
R62.8 (Other lack of expected normal physiological development)	88		
R62.9 (Lack of expected normal physiological development, unspecified)	30		
R63.0 (Anorexia)	7		
R63.3 (Feeding difficulties and mismanagement)	2		
R63.4 (Abnormal weight loss)	8		
R63.6 (Insufficient intake of food and water due to self neglect)	2		
R64 (Cachexia)	14		
R68.8 (Other specified general symptoms and signs)	20		
R91 (Abnormal findings on diagnostic imaging of lung)	2		
R99 (Other ill-defined and unspecified causes of mortality)	21		
S00-T98 (Injury, poisoning and certain other consequences of external causes)	170	0.1	0.1-0.1
S06.9 (Intracranial injury, unspecified)	3		
S12.9 (Fracture of neck, part unspecified)	1		
S14.1 (Other and unspecified injuries of cervical spinal cord)	1		

S19.9 (Unspecified injury of neck)	1
S31.0 (Open wound of lower back and pelvis)	1
S31.8 (Open wound of other and unspecified parts of abdomen)	1
S36.4 (Injury of small intestine)	1
S39.9 (Unspecified injury of abdomen, lower back and pelvis)	2
S72.0 (Fracture of neck of femur)	1
S72.9 (Fracture of femur, part unspecified)	1
S82.2 (Fracture of shaft of tibia)	1
S82.4 (Fracture of fibula alone)	1
S82.8 (Fractures of other parts of lower leg)	1
T08 (Fracture of spine, level unspecified)	1
T11.0 (Superficial injury of upper limb, level unspecified)	1
T13.9 (Unspecified injury of lower limb, level unspecified)	1
T14.9 (Injury, unspecified)	1
T17.8 (Foreign body in other and multiple parts of respiratory tract)	4
T17.9 (Foreign body in respiratory tract, part unspecified)	122
T18.9 (Foreign body in alimentary tract, part unspecified)	1
T68 (Hypothermia)	1
T71 (Asphyxiation)	1
T74.0 (Neglect or abandonment)	1
T75.1 (Drowning and nonfatal submersion)	2
T81.0 (Haemorrhage and haematoma complicating a procedure, not elsewhere classified)	1
T83.5 (Infection and inflammatory reaction due to prosthetic device, implant and graft in urinary system)	1
T83.8 (Other complications of genitourinary prosthetic devices, implants and grafts)	1
T85.0 (Mechanical complication of ventricular intracranial (communicating) shunt)	1
T85.7 (Infection and inflammatory reaction due to other internal prosthetic devices, implants and grafts)	2
T85.8 (Other complications of internal prosthetic devices, implants and grafts, not elsewhere classified)	2
T91.8 (Sequelae of other specified injuries of neck and trunk)	1
T93.1 (Sequelae of fracture of femur)	1
T94.0 (Sequelae of injuries involving multiple body regions)	1
T94.1 (Sequelae of injuries, not specified by body region)	1
T96 (Sequelae of poisoning by drugs, medicaments and biological substances)	1
T98.0 (Sequelae of effects of foreign body entering through natural orifice)	2

T98.1 (Sequelae of other and unspecified effects of external causes)	1		
T98.3 (Sequelae of complications of surgical and medical care, not elsewhere classified)	2		
V01-Y89 (External causes of morbidity and mortality)	202	0.1	0.1-0.1
W05 (Fall involving wheelchair)	1		
W06 (Fall involving bed)	1		
W19 (Unspecified fall)	1		
W65 (Drowning and submersion while in bath-tub)	1		
W74 (Unspecified drowning and submersion)	1		
W78 (Inhalation of gastric contents)	7		
W79 (Inhalation and ingestion of food causing obstruction of respiratory tract)	13		
W80 (Inhalation and ingestion of other objects causing obstruction of respiratory tract)	126		
W84 (Unspecified threat to breathing)	1		
X31 (Exposure to excessive natural cold (hypothermia))	1		
X59.0 (Exposure to unspecified factor causing fracture)	3		
X59.9 (Exposure to unspecified factor causing other and unspecified injury)	7		
Y06.9 (By unspecified person)	1		
Y34 (Unspecified event, undetermined intent)	1		
Y83.0 (Surgical operation with transplant of whole organ)	1		
Y83.2 (Surgical operation with anastomosis, bypass or graft)	4		
Y83.3 (Surgical operation with formation of external stoma)	11		
Y83.6 (Removal of other organ (partial) (total))	2		
Y83.8 (Other surgical procedures)	2		
Y83.9 (Surgical procedure, unspecified)	1		
Y84.6 (Urinary catheterization)	3		
Y84.8 (Other medical procedures)	2		
Y86 (Sequelae of other accidents)	6		
Y87.1 (Sequelae of assault)	1		
Y88.3 (Sequelae of surgical and medical procedures as the cause of abnormal reaction of the patient, or of later complication, without mention of misadventure at the time of the procedure)	4		n/a
